# Introductory Lecture on Materia Medica

**Published:** 1847-03

**Authors:** Robert M. Hoston


*Introductory Lecture to the course of* 1846–'7, *on Materia Medica, &c., in the Jefferson Medical College,* by Robert M. Hoston, M. D.

We have been put in possession of the introductory, bearing the above caption, and read it with more than usual interest. The writer, after making apology for men, frequently of no mean pretensions, deriding the medical profession, and asserting that it is undeserving of support; by observing that the advancement in late years, of all sciences and arts, have been so rapid that the community have become impatient of every thing that stops short of perfection; makes the following truthful observations, which might apply with equal force, to the dental as the medical profession;

"To the honor of the profession of the law, be it said, that the instances in which any of its distinguished members countenanced quackery in medicine are comparatively rare; their acquaintance with the laws of evidence enables them to detect the shallowness of the argument by which those of less disciplined minds are entrapped.

"I wish that it were in my power to say as much of the clergy—a class, whose influence on the mass of society is vastly greater, from the intimate relations that exist between them and their fellow-citizens in private and family circles—a class, whose lives are especially devoted to the best interests of their fellow-men, and among the members of which, in all ages, learning and science have had their ablest patrons, and most devoted cultivators; and yet, from clergymen, of various denominations, the vilest impostors and pretenders in medicine have received their most efficient support. It would seem as if the 'charity, which covereth a multitude of sins,' forbids them to suspect imposture in matters so sacred. Unaccustomed to the traffic and employments of ordinary life, they often know but little of the artifices too frequently practised to obtain success, and thus become the dupes of a too ready credulity. Artful appeals to their benevolence to lend a helping hand in dispensing relief to the sufferer, find with them a ready response, and 'out of the abundance of the heart the mouth speaketh,' hence, the certificates and testimonials of distinguished clergymen, which are found in the advertisement of every nostrum vender that are seen in the public newspapers of the day. If the statements which have appeared at sundry times, avouched by respectable clergymen, were true, all diseases should vanish from the earth, for we should have a remedy for every complaint.

"Have we not a right, then, to call upon such to withhold in future—to consider well before they lend their names and influence in such a cause—to abstain, lest they certify as true, what experience has shown will most probably turn out false? In my humble opinion, it is their solemn duty to take care that truth, humanity, and their own characters do not suffer by their presumptuously undertaking to decide questions of which they are almost necessarily ignorant."

The writer enters into an analysis of the claims upon the public confidence, of the various self-styled improvements in the healing art, which analysis is not in harmony with their pretensions.

The writer closes his lecture in a manner, indicating a mind deeply impressed with, not only the obligations, but the honor and dignity of his profession.

